# Supplementary material for: Confidence modulates the decodability of scene prediction during partially-observable maze exploration in humans
Source: Commun Biol. 2022 Apr 19;5:367. doi: 10.1038/s42003-022-03314-y (PMC9018866; doi:10.1038/s42003-022-03314-y)
Supplement: Supplementary file 2 — Supplementary Information [file 42003_2022_3314_MOESM2_ESM.pdf]

Supplementary Information for *Confidence modulates the decodability of scene prediction during partially-observable maze exploration in humans*

*Risa Katayama[1], †Wako Yoshida[2,3] and †Shin Ishii[1,4,5]

1. Graduate School of Informatics, Kyoto University, Kyoto, Kyoto, 606-8501 Japan
2. University of Oxford, Nuffield Department of Clinical Neuroscience, Oxford, OX3 9DU, UK
3. Department of Neural Computation for Decision-making, Advanced Telecommunications Research Institute International, Soraku-gun, Kyoto, 619-0288 Japan
4. Neural Information Analysis Laboratories, Advanced Telecommunications Research Institute International, Soraku-gun, Kyoto, 619-0288 Japan
5. International Research Center for Neurointelligence, the University of Tokyo, Bunkyo-ku, Tokyo, 113-0033 Japan

* Correspondence: katayama.risa.44n@st.kyoto-u.ac.jp
† These authors contributed equally to this work.

Supplemental Figures

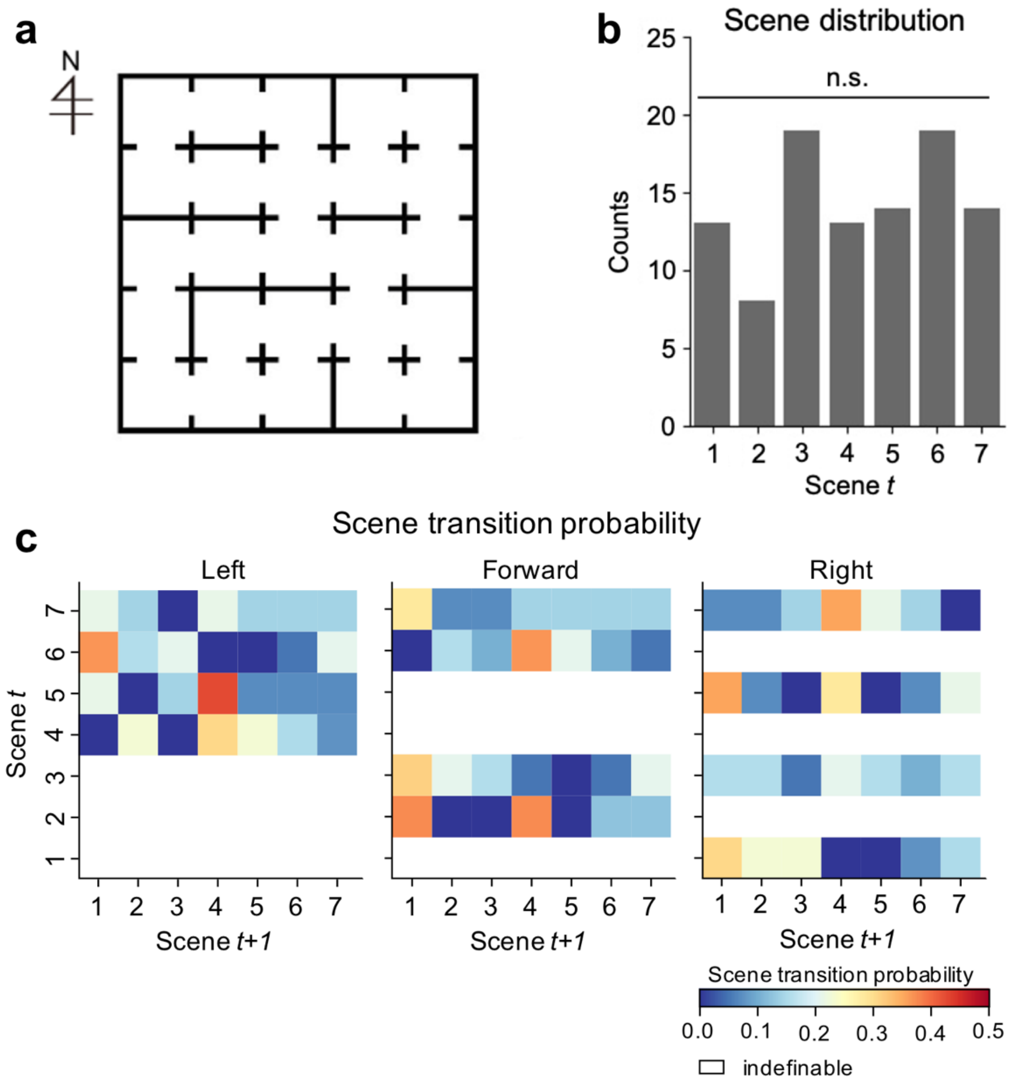

Supplementary Figure 1. Statistics for the 5 × 5 grid maze used in the experiment.

a) A 2D map of the maze. The maze consisted of a 5×5 grid in which each space has either an open (passable) or a closed (impassable) door on each of its four sides. During each trial, subjects were positioned within a square in the grid facing to in one of the cardinal directions: north, west, south or east.

b) The number of each type of scene (1–7) observable from all possible states in the maze (25 positions × 4 directions = 100 total states). No scene was uniquely associated with a single state, and the scene distribution was not significantly different from the uniform distribution ($X^2(6)=6.12$, $p=0.41$, n.s.: $p\geq 0.05$).

c) The scene transition (probability) matrices corresponding to each of the three possible movement actions: left, forward, and right. Each square within the heatmaps represents the observation probability of a scene, s_{t+1} (horizontal axis), followed by the action selected at a state with scene s_t

(vertical axis) in the previous trial: $P(s_{t+1}|s_t, a)$. Considering all combinations of scenes and actions, there are at least four types of upcoming scenes for which the observation probability is greater than zero.

These statistics suggest that state identification is not possible based on a single observed scene **(b)**, or on the combination of the previous scene and action **(c)** alone. Thus, in our maze exploration task, subjects were required to infer their current state based on the history of actions and observed scenes.

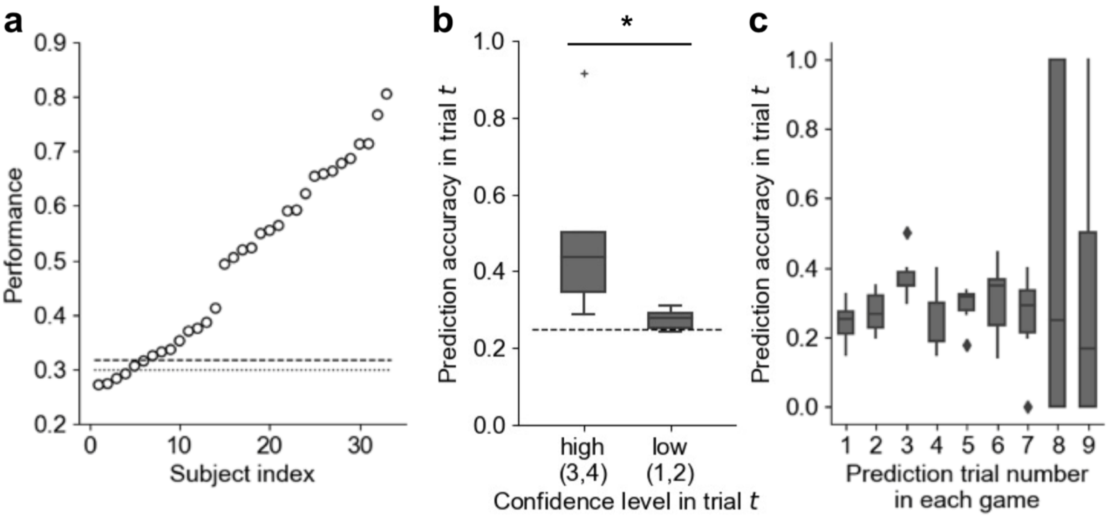

Supplementary Figure 2. Behavioral results of low performance subjects.

Six out of thirty-three subjects, whose scene prediction accuracy in the experimental task was not significantly higher than chance (one-sided z-test, $p \geq 0.01$), were excluded from the analyses.

a) The scene prediction performance of all 33 subjects, depicted in ascending order of the prediction accuracy. The dashed and dotted lines indicate the statistical threshold for one-sided z test to extract subjects who achieved significantly higher prediction accuracy than chance (dashed: α=0.01, dotted: α=0.05). In the analyses we used a stricter statistical threshold (α=0.01) than usual (α=0.05), but there was almost no difference in the number of the subjects that could be used for the analyses between the threshold (if α=0.05, 29 subjects).

b) For the excluded six subjects, the prediction accuracy was significantly higher when the subjects' confidence levels were high (level 3 or 4) (one-sided Wilcoxon signed-rank test, *: $p<0.05$), but the correlation was weaker than that for the 27 subjects included in the analyses (Figure 2b). The dashed line indicates the chance level (=0.25). Center lines of the box plots indicate the medians, boxes indicate the lower and upper quartiles, and the whiskers indicate 1.5× interquartile range (IQR). Cross-markers indicate the outliers.

c) Scene prediction accuracy of the six low performers, shown as a function of the number of successive prediction trials in each game. The prediction accuracy did not increase as the number of prediction trials performed in a single game increased (r=3.5×10^{-2}, p=0.77), while they were significantly correlated for the subject group with better prediction performance (**Figure 2c**). These results suggested a high possibility that the excluded subjects responded without scene prediction in the task.

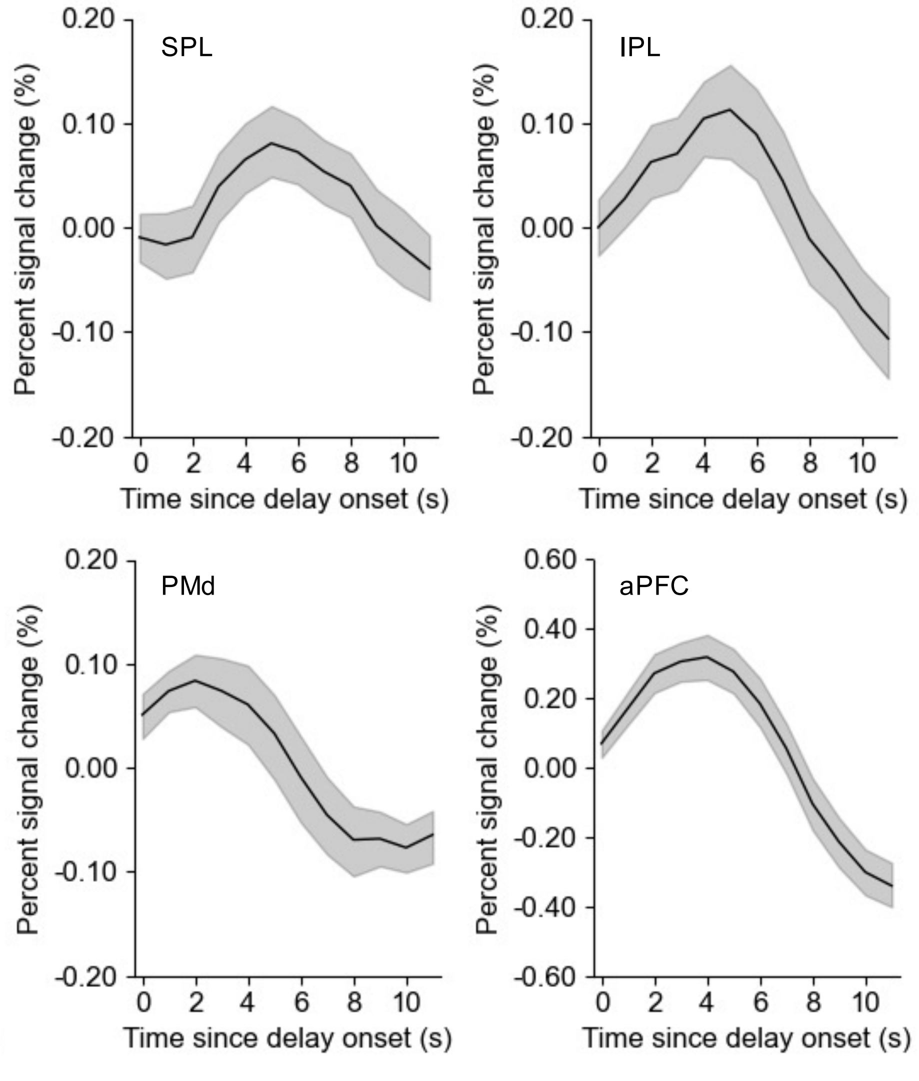

Supplementary Figure 3: Supplementary results of the univariate-level ROI analyses.
Time-series of the percent signal change of each ROI during the prediction trials**.** The neural responses were sampled at each TR (every 1 s) from the onset of the delay period (marked as 0 s). The lines and the colored area indicate the average and SEM over the 26 subjects included in the imaging and the decoding analyses.

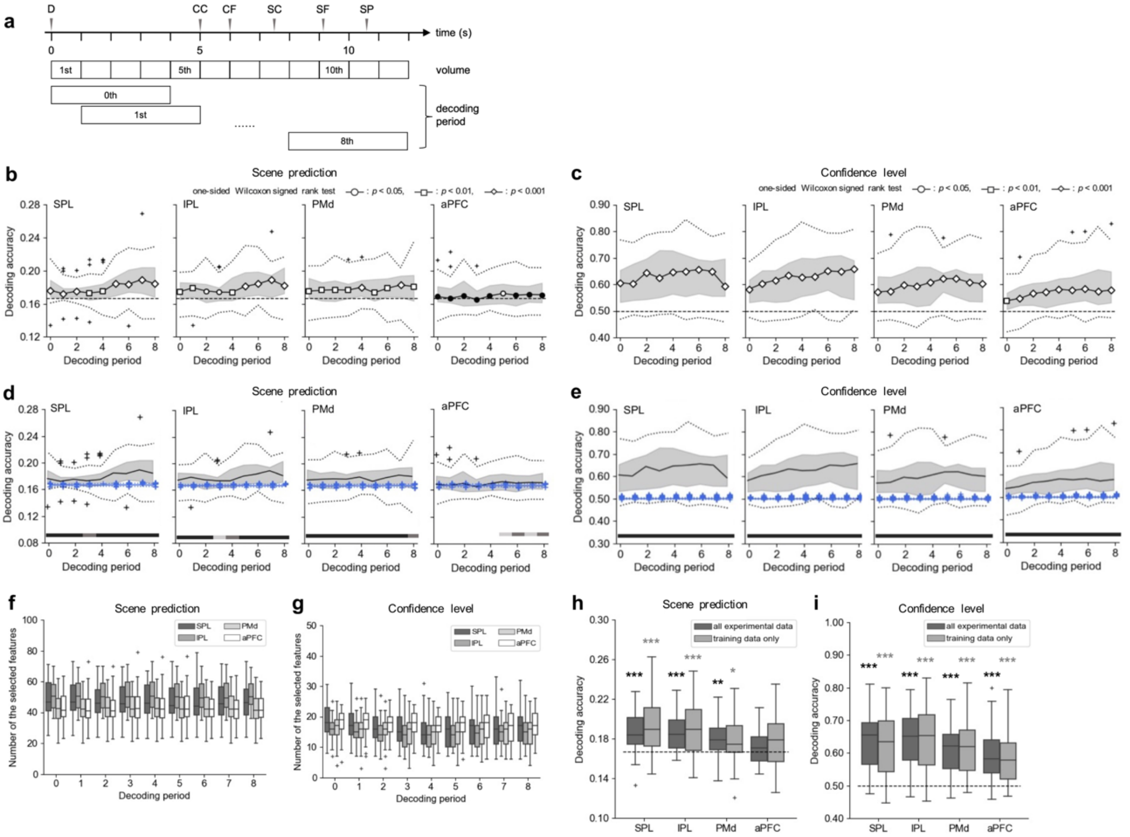

Supplementary Figure 4. Design of the time-series decoding analysis and supplementary results.

a) The design of the time-series decoding analysis and the temporal relationship of events in the prediction trial. In the time-series decoding analyses, the decoding result for the *t*-th decoding period was for the BOLD signal from *t* s to *t*+3 s (i.e., (*t*+1)-th to (*t*+4)-th volumes) after the onset of the delay period. The onset of each event since the delay onset (D) was: confidence choice (CC), 5.0s; confidence choice feedback (CF), 6.0s; scene choice, 7.5s; scene choice feedback (SF), 9.1s; next scene presentation (SP), 10.6s (mean for all prediction trials of 26 subjects included in the decoding analyses). Because the BOLD signal increases 4–6 s after its evoking event, the effect of the scene choice period, during which the subjects' predicted (chosen) scene is displayed, was not taken into account in our decoding analysis.

b,c) Time-series decoding results. Each panel shows the decoding accuracies of the scene prediction **(b)** and the subjects' reported confidence level about the scene prediction **(c)** within each ROI. The decoding accuracies were evaluated using leave-one-session-out (LOSO) cross validation (CV). The solid black lines represent the median, gray shaded areas indicate the range between the upper and lower quartiles, and the dotted lines indicate the range of 1.5×IQR. Cross-markers indicate the outliers. Significance was tested using a one-sided Wilcoxon signed-rank test compared to chance (dashed line). Unfilled markers indicate significant thresholds (circle: $p<0.05$, square: $p<0.01$,

diamond: $p<0.001$).

d,e) Results of statistical significance evaluation by a permutation test. Each plot shows the decoding accuracies for scene prediction (**d**, black) and its confidence level (**e**, black), and the corresponding permutation-based null distribution (blue). The solid lines represent the median, shaded areas indicate the range between the upper and lower quartiles, and the dotted lines indicate the range of 1.5×IQR. Cross-markers indicate the outliers. The differences between the permutation-based null distribution and the original decoding accuracies were evaluated with a one-sided Wilcoxon rank sum test, and the color of the horizontal line below the plots indicates the significance threshold (light gray: $p<0.05$, dim gray: $p<0.01$, black: $p<0.001$).

f,g) Numbers of relevant features selected by the SLR decoders. Each panel shows the numbers of features selected by the scene prediction decoders **(f)** and by the subject-reported confidence level decoders **(g)** using SLR. Each box extends from the lower to upper quartiles, with a horizontal line at the median. The whiskers show 1.5×IQR, and cross markers indicate the outliers.

h,i) Decoding results compared between different ROI selection methods. The accuracy of the scene prediction decoder **(h)** and the confidence decoder **(i)** was evaluated by leave-one-session-out cross-validation. In each fold, ROI selection was performed to ensure that there was no information leak when testing the validation data. For both decoders, the voxel activity patterns of the 6th decoding period was used. Each box extends from the lower to upper quartiles, with a horizontal line at the median. The whiskers show 1.5×IQR, and cross markers indicate the outliers. Significance was tested using a one-sided Wilcoxon signed rank test compared to chance (dotted line) (*: $p<0.05$, **: $p<0.01$, ***: $p<0.001$).

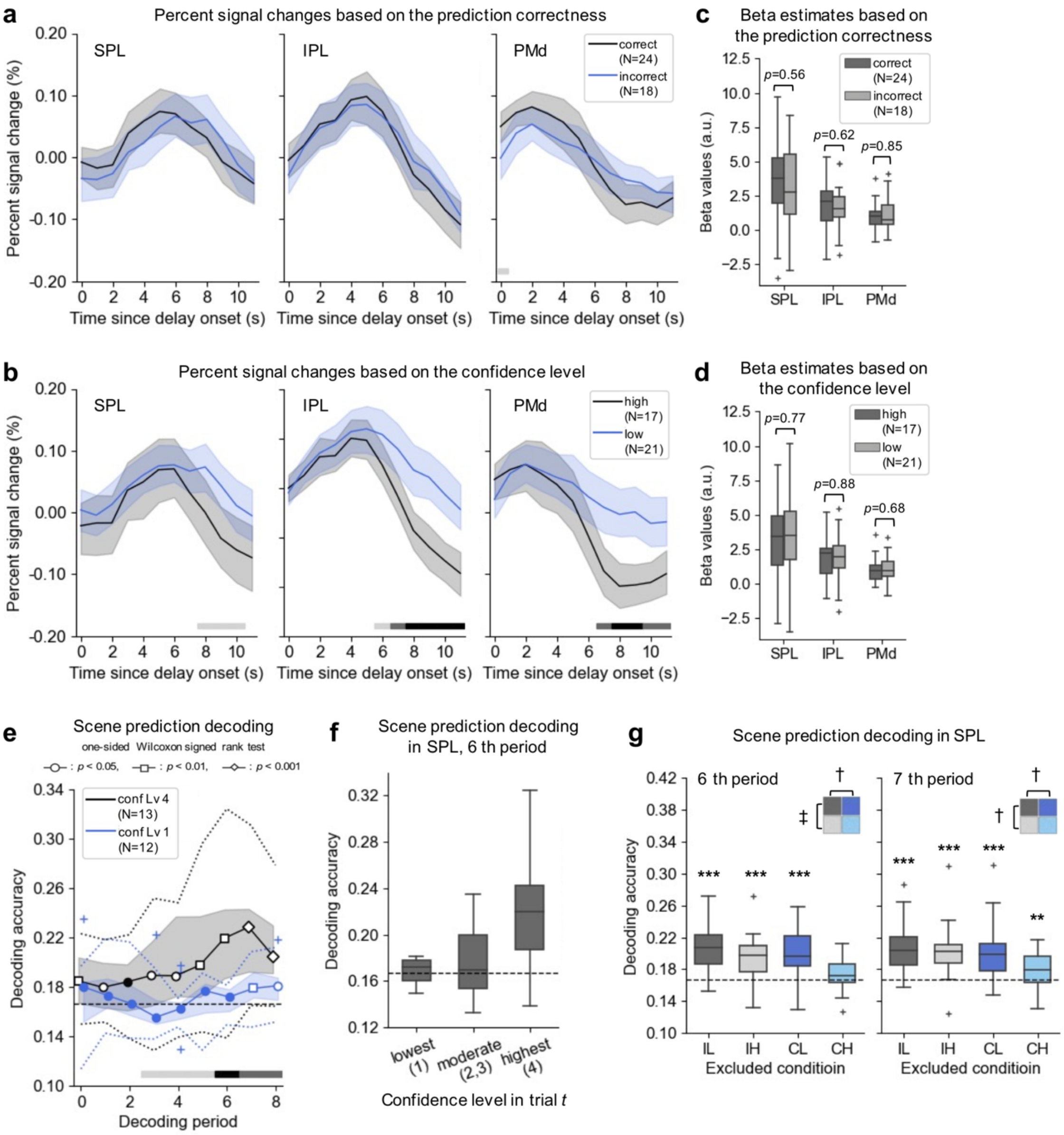

Supplementary Figure 5. Supplementary results of the conditional decoding analyses.

a-b) Time-series of the percent signal change of each ROI during the prediction trials when the data were categorized into two according to the prediction correctness **(a)** and the subject's reported confidence level **(b)**. The neural responses were sampled at each TR (every 1s) from the onset of the delay period (marked as 0 s). The lines and the colored area indicate the average and SEM over the subjects included in the corresponding conditional decoding analyses. The color of the horizontal line below the plots indicates the significant difference between the two categories of trials in each timepoint (two-sided Wilcoxon rank sum test, light gray: $p<0.05$, dim gray: $p<0.01$, black: $p<0.001$).

c-d) The beta value of each ROI in which the scene prediction was decodable, in comparison between correct and incorrect trials **(c)**, and high- and low-scene-confidence trials **(d)**. Each plot displays the

data of the subjects included in the corresponding conditional decoding analyses. There was no significant difference between the paired conditions for all three ROIs (two-sided Wilcoxon rank sum test).

e) Time-series decoding accuracies within SPL for scene prediction when the data were restricted to the most or least confidence trials (i.e., the trials with the confidence level 4 or 1). The accuracy was evaluated using leave-one-game-out (LOGO) CV. Significance was tested using one-sided Wilcoxon signed-rank test compared to chance (dashed line) and each unfilled marker indicated the significance threshold (square: $p<0.01$, circle: $p<0.05$). Differences in the decoding accuracy between the most and the least confidence levels were tested using a one-sided Wilcoxon rank sum test (light gray: $p<0.05$, dim gray: $p<0.01$, black: $p<0.001$).

f) Decoding accuracy for the scene prediction when the data was separated into three reported levels of confidence: the highest (confidence level 4), moderate (2 and 3), and lowest (1). The decoding accuracy monotonically and significantly increased as the confidence level increased ($r=0.52$, $p=3.8\times10^{-4}$). The accuracy was evaluated using LOGO CV. Note that this figure shows the results of the 6th decoding period as representative data. Similarly, there was a positive significant correlation between the scene prediction decodability in SPL and the confidence level, for the 3rd to 8th decoding period.

g) The decoding results with four types of scene prediction decoders, each of which was trained and tested with the trials from which one multiplicative condition was excluded to gain insights into the interaction effects between the prediction correctness and the scene prediction confidence. For example, 'IL'-excluded decoder (dimgray boxplot) was trained and tested by using the set of trials from which those of incorrect scene having been chosen with low confidence were excluded. The accuracy was evaluated using LOGO CV (one-sided Wilcoxon signed-rank test, **: $p<0.01$, ***: $p<0.001$), and the dashed line indicates the level of chance. Here we show the results with the activity patterns of SPL for the 6th and 7th decoding period described in the main text. Aligned rank transformation analyses of variance (ART-ANOVAs) revealed that the scene prediction decodability was significantly lower without the high-scene-confidence trials than without the low-scene-confidence trials, as well as without the correct trials than without the incorrect trials († : $p<0.05$, ‡: $p<0.01$). There was also no interaction effect between the correctness of the exclusive prediction and the confidence level (6th period, $F(1,96)=2.39$, $p=0.12$; 7th period, $F(1,96)=1.99$, $p=0.16$). These results suggest that the neural representation of prediction in SPL is more distinctive when the upcoming scene is correctly predicted and when the upcoming scene is predicted with high confidence.

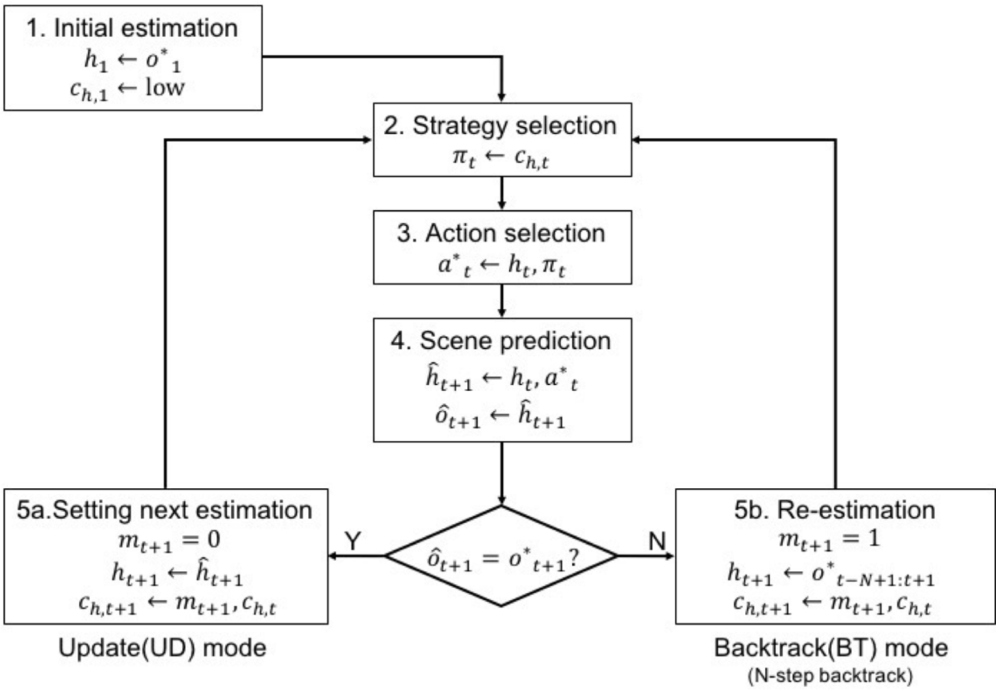

Supplementary Figure 6. Subject behavioral model.

Block diagram of the hidden Markov model (HMM), as a subject behavioral model with a latent confidence level. According to the model, the subjects have three kinds of cognitive state variables—the operant state (m_t), the state inference (h_t). and the confidence level for the state inference (c_h)—all of which are objectively estimated based on the sequence of observed scenes (o_t^*) and action (a_t^*). π_t denotes the action selection strategy. The number assigned to each block (i.e., 1–5b) corresponds to a process step described in the Methods, ***Behavioral model based on a hidden Markov model***.

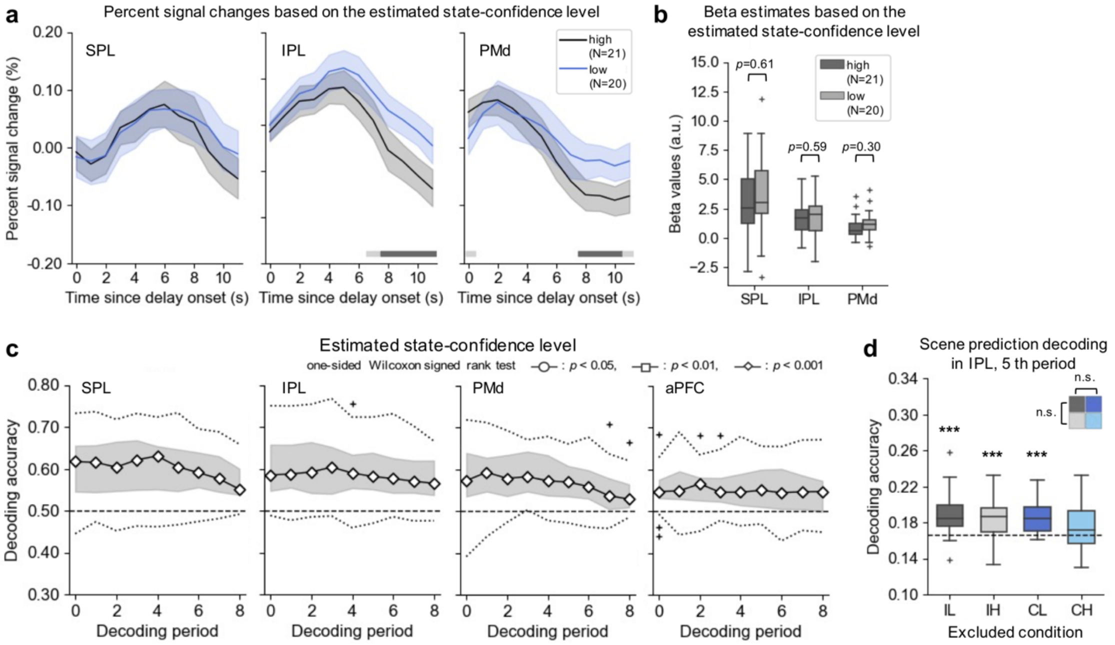

Supplementary Figure 7. Supplementary results of the model-based decoding analyses.

a) Time-series of the percent signal change of each ROI during the prediction trials when the data were categorized into two according to the estimated state-confidence level. The neural responses were sampled at each TR (every 1 s) from the onset of the delay period (marked as 0 s). The lines and the colored area indicate the average and SEM over the subjects included in the corresponding conditional decoding analyses. The color of the horizontal line below the plots indicates the significant difference between the two categories of trials in each timepoint (two-sided Wilcoxon rank sum test, light gray: $p<0.05$, dim gray: $p<0.01$, black: $p<0.001$).

b) The beta value of each ROI in which the scene prediction was decodable, in comparison between high- and low-state-confidence trials. Each plot displays the data of the subjects included in the corresponding conditional decoding analyses. Center lines of the box plots indicate the medians, boxes indicate the lower and upper quartiles, and the whiskers indicate 1.5×IQR. Cross-markers indicate the outliers. There was no significant difference between the paired conditions for all three ROIs (two-sided Wilcoxon rank sum test).

c) Time-series decoding results. Each panel shows the decoding accuracies at each ROI of the state-confidence level estimated by the subject behavioral model. The decoding accuracies were evaluated using LOSO CV. The solid black lines represent the median, gray shaded areas indicate the range between the upper and lower quartiles, and the dotted lines indicate the range of 1.5×IQR. Cross markers indicate the outliers. Significance was tested using a one-sided Wilcoxon signed-rank test compared to chance (dashed line). Unfilled markers indicate significant thresholds (circle: $p<0.05$, square: $p<0.01$, diamond: $p<0.001$).

d) The decoding results with four types of scene prediction decoders, each of which was trained and tested with the trials from which one multiplicative condition was excluded (see also **Supplementary Figure 5g**), to gain insights into the interaction effects between the correctness and the state-confidence. The accuracies ware evaluated using LOGO CV (one-sided Wilcoxon signed-rank test, *: p<0.05, **: p<0.01, ***: p<0.001), and the dashed line indicates the level of chance. Here we present the results with the activity patterns of IPL in the 5th period, in which the difference in the scene prediction decodability was highly significant between the high- and low-state-confidence trials. The ART-ANOVA showed no main effect of excluding data based on both prediction correctness ($F(1,97)=1.60$, $p=0.21$) and the confidence level ($F(1,97)=2.80$, $p=9.8\times10^{-2}$). However, the IPL scene prediction decoder failed to show higher accuracy than chance only when correct and high-state-confidence trials were excluded. This result suggests that the neural representation of prediction in IPL is more distinctive when the upcoming scene is predicted correctly with high-state confidence.

Supplemental Tables

Supplementary Table 1. Peak voxels in areas exhibiting significant activity during the first four seconds of the delay period during prediction trials.

Region of activation	L/R	BA	MNI coordinates			z-value	No. of voxels
			x	y	z		
Superior parietal lobule	L/R	7	-8	-70	46	5.62	1063
Inferior parietal lobule	L	40	-38	-46	36	5.46	1762
Inferior parietal lobule	R	40	46	-48	44	4.59	924
Middle frontal gyrus	L	6	-24	-2	54	4.57	660
Middle frontal gyrus	L	10	-30	50	0	5.33	497

Supplementary Table 2. The correlation between the number of samples and the decoding accuracy for each scene.

	SPL	IPL	PMd	aPFC
0th	$r=-9.1\times10^{-2}$, $p=0.26$	$r=-0.11$, $p=0.18$	$r=-0.14$, $p=7.4\times10^{-2}$	$r=-5.2\times10^{-2}$, $p=0.52$
1st	$r=-0.15$, $p=7.0\times10^{-2}$	$r=-7.3\times10^{-2}$, $p=0.37$	$r=-0.13$, $p=0.10$	$r=-4.1\times10^{-2}$, $p=0.61$
2nd	$r=-0.13$, $p=0.11$	$r=-0.15$, $p=5.6\times10^{-2}$	$r=-0.19$, $p=2.1\times10^{-2}$	$r=-9.6\times10^{-2}$, $p=0.23$
3rd	$r=-0.11$, $p=0.16$	$r=-7.6\times10^{-2}$, $p=0.34$	$r=-0.13$, $p=0.10$	$r=-0.14$, $p=7.1\times10^{-}$
4th	$r=-5.4\times10^{-2}$, $p=0.51$	$r=-3.8\times10^{-2}$, $p=0.64$	$r=-0.16$, $p=5.1\times10^{-2}$	$r=-3.2\times10^{-2}$, $p=0.69$
5th	$r=-7.2\times10^{-2}$, $p=0.37$	$r=-4.3\times10^{-2}$, $p=0.59$	$r=-9.9\times10^{-2}$, $p=0.22$	$r=-2.6\times10^{-2}$, $p=0.74$
6th	$r=-3.4\times10^{-2}$, $p=0.67$	$r=-6.2\times10^{-2}$, $p=0.44$	$r=-5.6\times10^{-2}$, $p=0.49$	$r=-4.9\times10^{-2}$, $p=0.55$
7th	$r=-8.9\times10^{-2}$, $p=0.27$	$r=-0.13$, $p=0.12$	$r=-0.16$, $p=4.3\times10^{-2}$	$r=-2.9\times10^{-2}$, $p=0.72$
8th	$r=-0.11$, $p=0.18$	$r=-0.19$, $p=1.5\times10^{-2}$	$r=-0.18$, $p=2.5\times10^{-2}$	$r=-7.6\times10^{-2}$, $p=0.35$

Supplementary Table 3. Estimated parameters for each subject using the hidden Markov model-based behavioral model. Our HMM-based behavioral model (**Supplementary Figure 6**) had five parameters: the optimal action selection probability for the forward-dominant strategy (α), that for the efficient-exploration strategy (β), the probability of changing from low to high confidence level in update mode ($p_{L\rightarrow H}$), the probability of changing from high to low confidence level in backtrack mode ($p_{H\rightarrow L}$), and the number of past observations used for the backtrack re-estimation

(n). The parameter ranges were predetermined: $p_{L\to H}$ and $p_{H\to L}$ ranged from 0.1 to 0.9, α ranged from 0.5 to 0.9, and β ranged from 2/3 to 0.9. Here, the lower bound for β was set as $2/3$ because the probability of choosing an optimal action should always be larger than that of choosing a non-optimal action (i.e., when there were three open doors, two of which were optimal). n was equal to either 1, 2 or 3. This table shows the parameters for each individual, estimated such as to minimize the negative log evidence (equation (10) in the main text).

**Subject**	$\boldsymbol{\alpha}$	$\boldsymbol{\beta}$	$\boldsymbol{p_{L\to H}}$	$\boldsymbol{p_{H\to L}}$	$\boldsymbol{n}$
1	0.86	0.75	0.22	0.21	3
2	0.83	0.75	0.39	0.12	3
3	0.69	0.74	0.71	0.20	3
4	0.78	0.76	0.16	0.20	2
5	0.53	0.78	0.23	0.19	1
6	0.65	0.88	0.33	0.28	2
7	0.51	0.73	0.65	0.25	3
8	0.87	0.77	0.58	0.13	3
9	0.67	0.89	0.30	0.79	2
10	0.78	0.83	0.27	0.15	2
11	0.85	0.75	0.61	0.12	1
12	0.78	0.84	0.33	0.15	3
13	0.88	0.78	0.32	0.16	3
14	0.52	0.74	0.34	0.18	2
15	0.58	0.82	0.21	0.17	2
16	0.85	0.86	0.46	0.14	1
17	0.86	0.70	0.23	0.41	3
18	0.88	0.81	0.54	0.13	1
19	0.85	0.79	0.37	0.12	1
20	0.52	0.69	0.59	0.41	3
21	0.86	0.81	0.19	0.18	2
22	0.88	0.81	0.52	0.14	1
23	0.77	0.75	0.15	0.64	2
24	0.69	0.68	0.35	0.78	3
25	0.76	0.78	0.15	0.74	1
26	0.76	0.88	0.35	0.27	3
27	0.88	0.85	0.47	0.19	2

Supplementary Table 4. Bayesian model comparison results. To test the credibility of our HMM-based behavioral model with a latent confidence level (**Supplementary Figure 6**), here called the strategy-switching model (SS model), we compared it with two alternative models that incorporated a single strategy: A forward-dominant (FD) model, in which subjects were assumed to take a forward move if allowable, or to take an info-max move if the forward move was not allowed, and an efficient-exploration (EE) model, in which subjects were assumed to explore the maze most efficiently (i.e., preferentially moving to previously unexplored grid spaces, regardless of the state estimate confidence level). The FD and EE models corresponded to aspects of the SS model in which the confidence level was always low and always high, respectively. This means that they did not depend on the subjective value of the confidence level. Both FD and EE models included two parameters: the optimal action selection probability (α for FD; β for EE) and the number of past observations used in the backtrack mode (n). When comparing these three models, we used the parameter values estimated with each model. As can be seen in this table, Bayesian model selection in the random effect analysis showed that SS model was significantly better than the FD and EE models in terms of negative log-evidence and AIC.

**Criterion**	**Model**	**Model expected probability**	**Model exceedance probability**	**Protected exceedance probability**	**Subject-wise mean ± SD**
Negative Log-evidence	SS	0.66	0.99	0.98	126.5±33.7
	FD	0.25	1.3×10^{-2}	1.8×10^{-2}	131.0±42.9
	EE	8.7×10^{-2}	1.0×10^{-4}	4.5×10^{-3}	155.2±18.3
AIC	SS	0.43	0.56	0.43	263.0±67.4
	FD	0.40	0.43	0.38	266.1±85.8
	EE	0.17	8.2×10^{-3}	0.20	314.4±36.6
BIC	SS	0.22	3.3×10^{-2}	0.24	283.3±67.7
	FD	0.49	0.87	0.50	274.2±85.9
	EE	0.29	9.5×10^{-2}	0.26	322.5±36.8
