# Supplementary material for: Confidence modulates the decodability of scene prediction during partially-observable maze exploration in humans
Source: Commun Biol. 2022 Apr 19;5:367. doi: 10.1038/s42003-022-03314-y (PMC9018866; doi:10.1038/s42003-022-03314-y)
Supplement: Supplementary file 4 — Reporting Summary [file 42003_2022_3314_MOESM4_ESM.pdf]

nature portfolio

Corresponding author(s): Risa Katayama

Last updated by author(s): Mar 17, 2022

Reporting Summary

Nature Portfolio wishes to improve the reproducibility of the work that we publish. This form provides structure for consistency and transparency in reporting. For further information on Nature Portfolio policies, see our Editorial Policies and the Editorial Policy Checklist.

Statistics

For all statistical analyses, confirm that the following items are present in the figure legend, table legend, main text, or Methods section.

n/a	Confirmed	
☐	☒	The exact sample size (*n*) for each experimental group/condition, given as a discrete number and unit of measurement
☐	☒	A statement on whether measurements were taken from distinct samples or whether the same sample was measured repeatedly
☐	☒	The statistical test(s) used AND whether they are one- or two-sided<br>*Only common tests should be described solely by name; describe more complex techniques in the Methods section.*
☒	☐	A description of all covariates tested
☒	☐	A description of any assumptions or corrections, such as tests of normality and adjustment for multiple comparisons
☐	☒	A full description of the statistical parameters including central tendency (e.g. means) or other basic estimates (e.g. regression coefficient) AND variation (e.g. standard deviation) or associated estimates of uncertainty (e.g. confidence intervals)
☐	☒	For null hypothesis testing, the test statistic (e.g. *F*, *t*, *r*) with confidence intervals, effect sizes, degrees of freedom and *P* value noted<br>*Give P values as exact values whenever suitable.*
☒	☐	For Bayesian analysis, information on the choice of priors and Markov chain Monte Carlo settings
☒	☐	For hierarchical and complex designs, identification of the appropriate level for tests and full reporting of outcomes
☐	☒	Estimates of effect sizes (e.g. Cohen's *d*, Pearson's *r*), indicating how they were calculated

Our web collection on statistics for biologists contains articles on many of the points above.

Software and code

Policy information about availability of computer code

Data collection	Data collected with the custom code using Psychopy3 (Peirce et al., 2019).
Data analysis	The behavioral analyses were done with Matlab R2017a and R 4.0.4. The imaging analysis was done with SPM12 (Wellcome Department of Cognitive Neurology, London, UK), and the decoding analyses were done using the Brain Decoding Toolbox (Kamitani and Tong, 2005). For the model-based analyses, we used the custom codes for the computational model of subjects' exploration behaviors.

For manuscripts utilizing custom algorithms or software that are central to the research but not yet described in published literature, software must be made available to editors and reviewers. We strongly encourage code deposition in a community repository (e.g. GitHub). See the Nature Portfolio guidelines for submitting code & software for further information.

Data

Policy information about availability of data

All manuscripts must include a data availability statement. This statement should provide the following information, where applicable:

- Accession codes, unique identifiers, or web links for publicly available datasets
- A description of any restrictions on data availability
- For clinical datasets or third party data, please ensure that the statement adheres to our policy

The source data underlying the main figures are provided as Supplementary Data 1. All data supporting the main findings is also available via the open source repository Zenodo (https://doi.org/10.5281/zenodo.6364553).

Field-specific reporting

Please select the one below that is the best fit for your research. If you are not sure, read the appropriate sections before making your selection.

☒ Life sciences ☐ Behavioural & social sciences ☐ Ecological, evolutionary & environmental sciences

For a reference copy of the document with all sections, see nature.com/documents/nr-reporting-summary-flat.pdf

Life sciences study design

All studies must disclose on these points even when the disclosure is negative.

Sample size	No statistical methods was used to predetermine the sample size, but the sample size for our analyses was comparable to those generally employed in the field.
Data exclusions	Six subjects whose scene prediction accuracy in the experimental task was not significantly higher than chance (one-sided z-test, p>0.01, see also Supplementary Figure 2) were excluded from analyses. Another subject was also excluded from imaging and decoding analyses due to his/her large head motion (more than 5% of TRs at Framewise Displacement threshold 0.5 mm).
Replication	n/a
Randomization	Not relevant due to one experimental group
Blinding	Not relevant due to one experimental group

Reporting for specific materials, systems and methods

We require information from authors about some types of materials, experimental systems and methods used in many studies. Here, indicate whether each material, system or method listed is relevant to your study. If you are not sure if a list item applies to your research, read the appropriate section before selecting a response.

Materials & experimental systems

n/a	Involved in the study	
☒	☐	Antibodies
☒	☐	Eukaryotic cell lines
☒	☐	Palaeontology and archaeology
☒	☐	Animals and other organisms
☐	☒	Human research participants
☒	☐	Clinical data
☒	☐	Dual use research of concern

Methods

n/a	Involved in the study	
☒	☐	ChIP-seq
☒	☐	Flow cytometry
☐	☒	MRI-based neuroimaging

Human research participants

Policy information about studies involving human research participants

Population characteristics	Thirty-three healthy subjects (aged 20–32 years; four females) participated in this study.
Recruitment	Participants were recruited at Kyoto University and Advanced Telecommunications Research Institute International. We did not have any self-selection bias.
Ethics oversight	This study was approved by the ethical committees of the Advanced Telecommunications Research Institute International, Japan, and the Graduate School of Informatics, Kyoto University, Japan.

Note that full information on the approval of the study protocol must also be provided in the manuscript.

Magnetic resonance imaging

Experimental design

Design type	Task; event-related design
Design specifications	Each subject performed up to 40 total games (38.2±4.2 games), which were divided into three or four sessions. Volume acquisition was synchronized with the onset of the fixation cross presentation (4-6 s) in each prediction trial.

Behavioral performance measures | We recorded the subjects' chosen scenes as their upcoming scene prediction, the reported confidence levels about their scene prediction and the scene-choice reaction times.

Acquisition

Imaging type(s) | Structural and functional images

Field strength | 3T

Sequence & imaging parameters | A 3.0-Tesla Siemens MAGNETOM Prisma fit scanner (Siemens Healthineers, Erlangen, Germany) with a standard 64 channel phased array head coil was used for image acquisition. We acquired interleaved T2*-weighted echo-planar images (EPIs) (TR, 1000 ms; TE, 30 ms; flip angle, 50°; matrix size, 100×100; field of view, 200×200; voxel size, 2×2×2.5 mm; number of slices, 66). Volume acquisition was synchronized with the onset of the fixation cross presentation during each prediction trial. We also acquired whole-brain high-resolution T1-weighted structural images using a standard MPRAGE sequence (TR, 2250 ms; TE, 3.06 ms; flip angle, 9°; field of view, 256×256; voxel size, 1×1×1 mm).

Area of acquisition | a whole brain

Diffusion MRI ☐ Used ☒ Not used

Preprocessing

Preprocessing software | Imaging data were preprocessed using SPM12 (Wellcome Department of Cognitive Neurology, London, UK).

Normalization | For each subject, all functional images were aligned to the first image as a reference, coregistered to the individual high-resolution anatomical image, normalized into an MNI template, and spatially smoothed with a Gaussian kernel filter (FWHM, 8 mm).

Normalization template | We used an MNI template.

Noise and artifact removal | Six motion correction parameters for each session produced during realignment were used as nuisance variables.

Volume censoring | We did not apply volume censoring.

Statistical modeling & inference

Model type and settings | To find cortical voxels that were both significantly and commonly activated during predicting the upcoming scene view (the first 4 s of the delay period in the prediction trials) across all subjects, we performed univariate general linear model (GLM) analysis. Our GLM included seven regressors coding for onsets and durations of events in each session: action selection and moving scenery in the action trials, delay period, confidence evaluation, feedback for confidence evaluation, predicted scene choice, and scene choice feedback in the prediction trials. For the delay period (regressor-of-interest), although the time length varied trial-by-trial, we modeled it as a boxcar function for 4 s (the minimum duration of the delay period). These regressors were convolved with a hemodynamic response function (HRF). Additionally, motion correction parameters produced during realignment were included as nuisance variables for the GLM. The first-level GLM analysis was performed using the contrast vector whose element was 1 for the regressor-of-interests, and 0 otherwise. We performed a group random effect analysis using anatomically-localized cerebral cortex.

Effect(s) tested | see above.

Specify type of analysis: ☒ Whole brain ☐ ROI-based ☐ Both

Statistic type for inference (See Eklund et al. 2016) | voxel-wise

Correction | FWE-corrected at the cluster level

Models & analysis

n/a	Involved in the study
☒	☐ Functional and/or effective connectivity
☒	☐ Graph analysis
☐	☒ Multivariate modeling or predictive analysis

Multivariate modeling and predictive analysis | Voxel activity patterns during the delay period were used to decode both scene prediction and confidence. All fMRI data were spatially realigned, normalized, and smoothed with a Gaussian kernel (8 mm FWHM), and preprocessed with linear trend removal and z-score normalization for each voxel in every run over the time-series, but not convolved with HRF.
In the time-series decoding analysis, the decoder at each time t in the time course (t-th decoding period) used as its input the voxel-wise BOLD signal intensities averaged over four volumes corresponding to t s to t +3 s (i.e., (t+1)-th to (t+4)-th scan volumes) after the onset of the delay period (Supplementary Figure 4a). We limited the time-series decoding analysis up to the 8th period in order to cover 4–6 s after the delay

onset.
We used a sparse logistic regression (SLR; Yamashita et al., 2008) as a supervised learning algorithm for both the scene prediction and confidence decoders.
To deal with unbalanced training data sets (Figure 1c), we used an undersampling method to assign an equal number of samples to each label. To assess decoder accuracies, we used Leave-one-session-out (LOSO) cross-validatiton (CV), and when evaluating the decoders with the trials divided into two categories according to confidence or correctness, we used Leave-one-game-out (LOGO) CV. In each fold of the LOSO and LOGO validations, we repeated the following procedure 100 times to account for fluctuations in accuracy due to selected samples in the undersampling phase: random under-sampling from the training data set, training the decoder, and evaluating the decoder's accuracy.
